# Bezold’s Abscess Secondary to a Subclinical Mastoid Infection: Diagnostic Challenges and Literature Review

**DOI:** 10.7759/cureus.94544

**Published:** 2025-10-14

**Authors:** Shingo Umemoto, Sonoka Takakura, Takashi Hirano

**Affiliations:** 1 Department of Otorhinolaryngology - Head and Neck surgery, Faculty of Medicine, Oita University, Yufu, JPN

**Keywords:** bezold’s abscess, immunocompromised host, masked mastoiditis, mastoidectomy, otitis media complication

## Abstract

Bezold’s abscess is a rare but serious extracranial complication of otitis media, typically resulting from erosion of the mastoid tip. We report the case of a 34-year-old male with a two-month history of recurrent right-sided otalgia, occurring two to three times and each treated with short courses of oral antibiotics, without any ear discharge, who presented with a progressively enlarging, painful, right cervical swelling and low-grade fever. The recurrent mild otologic symptoms, likely the result of repeated antibiotic exposure, delayed recognition of the more serious underlying mastoiditis. Imaging revealed erosion of the mastoid tip and opacification of the mastoid air cells. According to the clinical and radiological findings, the condition was consistent with *masked mastoiditis*, a subclinical mastoid infection in which inflammation remains confined to the mastoid cavity due to blockage of the aditus ad antrum, often following partial suppression of acute symptoms by antibiotics. The absence of tympanic membrane perforation or obvious otorrhea further delayed recognition until complications developed. Surgical drainage and mastoidectomy were curative. Notably, this episode also led to the incidental discovery of previously undiagnosed diabetes mellitus, highlighting the role of impaired host immunity in disease progression. This case underscores the diagnostic challenges of masked mastoiditis and illustrates the need to consider it as an underlying pathology in unexplained deep neck infections.

## Introduction

Bezold’s abscess is a rare but potentially life-threatening complication of otitis media characterized by the spread of infection from the mastoid tip into the deep cervical spaces. The condition is seldom seen in modern clinical practice because of effective antibiotic therapies [1]. However, atypical presentations, such as in patients without ear symptoms, may delay diagnosis. One such atypical presentation, known as *masked mastoiditis*, can progress silently despite the decreased incidence of mastoiditis in the antibiotic era, and may only become apparent after complications develop, sometimes resulting in life-threatening outcomes [2-5]. In this report, we present a patient with Bezold’s abscess in whom masked mastoiditis was retrospectively identified as the underlying pathology in the absence of obvious otologic symptoms. In addition, the patient was newly diagnosed with diabetes mellitus during the disease course, highlighting the immunologic vulnerability associated with metabolic disorders. Such host factors, including impaired immune function due to diabetes mellitus, may predispose individuals to atypical presentations and facilitate disease progression.

## Case presentation

History of present illness

**Figure 1:**
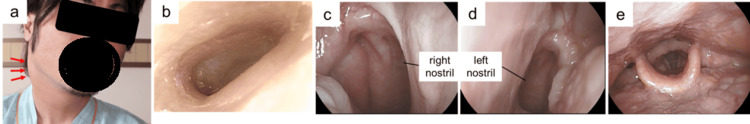
Initial clinical presentation. (a) Neck examination: A tender, fluctuant mass is noted in the upper anterior cervical region along the sternocleidomastoid muscle (arrows). (b) Endoscopic right-sided otoscopy reveals a thickened, opaque tympanic membrane with an erythematous external auditory canal. (c-e) Endoscopic pharyngeal and laryngeal views show no evidence of mucosal inflammation or swelling in the nasopharynx (c, d) or in the hypopharynx and larynx (e).

A 34-year-old man presented to the emergency department with right-sided otalgia and a progressively enlarging, painful swelling on the right side of the neck. He reported a two-month history of recurrent right ear pain, occurring two to three times. Each episode had been treated as otitis media with short courses of oral antibiotics. Despite temporary improvement after each short course of antibiotics, the symptoms recurred, and the neck swelling gradually worsened, prompting his emergency visit. He denied otorrhea or recent upper respiratory symptoms. Physical examination revealed a tender, elastic, fluctuant mass measuring approximately 8 cm in its greatest dimension in the upper anterior cervical region along the sternocleidomastoid muscle (Figure 1a). The mass showed limited mobility on palpation, and no additional cervical masses were detected. Otoscopic examination demonstrated a thickened, opaque right tympanic membrane with an erythematous external auditory canal (Figure 1b). Examination of the pharynx and larynx revealed no mucosal inflammation or swelling (Figures 1c-1e). The laboratory results suggested the presence of a bacterial infection, with an elevated neutrophil-predominant white blood cell count (19,430 /µL, neutrophils 84.3%) and a high serum C-reactive protein (17.57 mg/dL) (Table 1).

**Table 1:** Laboratory data on admission and at discharge. Laboratory findings showed marked inflammation and hyperglycemia on admission, both of which improved postoperatively and after endocrinological management. N/A, not applicable

Parameter	On admission (operative day)	Postoperative day 15 (discharge)	Normal range
White blood cell count (/µL)	19,430	6,750	3,100-8,400
Neutrophils (%)	84.3	53.5	41-72
C-reactive protein (mg/dL)	17.57	0.06	0.00-0.14
Fasting blood sugar (mg/dL)	196	95	73-109
HbA1c (%)	7.4	N/A	4.9-6.0

Imaging findings

**Figure 2:**
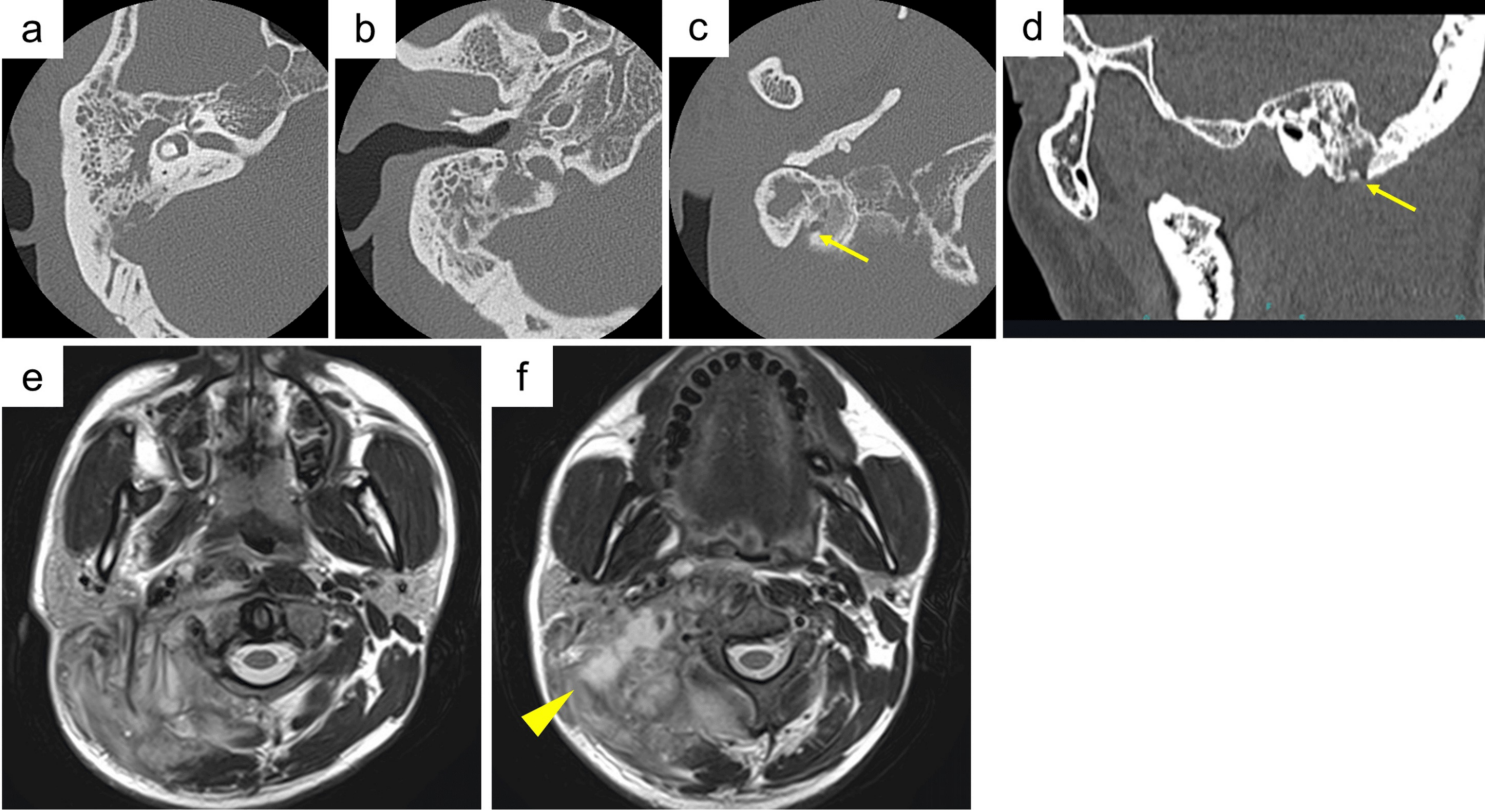
Imaging findings on presentation. (a) Axial target CT of the temporal bone shows complete opacification of the left mastoid air cells. (b) Opacification of the middle ear is seen, with no obvious destruction of the ossicles. (c) CT image demonstrating bone destruction at the mastoid tip. (d) Sagittal CT confirming a focal bony defect at the mastoid tip. (e, f) Axial T2-weighted MRI shows swelling of the posterior cervical muscles (e) and signal changes suggestive of abscess formation (f). The arrow in panels (c) and (d) indicates bony destruction of the mastoid tip, and the arrowhead in panel (e) indicates the abscess cavity. CT, computed tomography; MRI, magnetic resonance imaging

Computed tomography (CT) and magnetic resonance imaging (MRI) of the head and neck were performed to comprehensively evaluate both bony and soft tissue structures. Because the patient had a history of bronchial asthma, contrast-enhanced CT was avoided to minimize the risk of asthma exacerbation. Non-contrast CT provided a detailed assessment of mastoid air-cell opacification (Figures 2a-2b) and bony erosion at the mastoid tip (Figures 2c-2d), whereas MRI was essential for delineating the extent of abscess formation and evaluating soft tissue inflammation (Figures 2e-2f). Together, these imaging modalities confirmed the diagnosis of Bezold’s abscess secondary to masked mastoiditis.

Surgical findings

**Figure 3:**
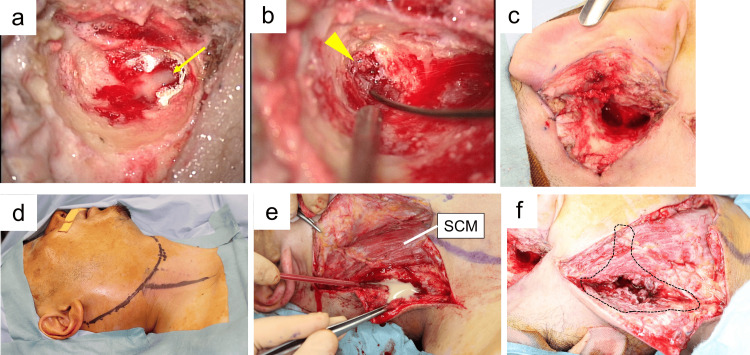
Operative findings. (a) Intraoperative microscopic view during mastoidectomy showing purulent discharge from the mastoid tip (arrow). (b) The mastoid antrum is filled with granulation tissue (arrowhead), which was removed. (c) Macroscopic view after mastoidectomy. (d) Skin incision line for cervical abscess drainage. (e) Dissection along the posterior aspect of the sternocleidomastoid muscle exposed the abscess cavity, which was opened and debrided. (f) Post-debridement view after thorough irrigation. The dotted line indicates the outer border of the abscess cavity. SCM, sternocleidomastoid muscle

The patient underwent prompt surgical drainage of the neck abscess, followed by a cortical mastoidectomy via postauricular incision. When the mastoid tip was reached, a purulent discharge was encountered (Figure 3a). The mastoid cavity was filled with granulation tissue, which was meticulously removed (Figures 3b-3c). Subsequently, a cervical incision was made (Figure 3d). Dissection along the sternocleidomastoid muscle exposed the abscess cavity, which was opened (Figure 3e). The wound was irrigated copiously (Figure 3f), and an open drain was placed before closure. Intraoperative findings confirmed purulent material within the mastoid cavity without substantial middle ear involvement. Samples from both the mastoid cavity and the cervical abscess were submitted for bacterial culture; however, no organisms were isolated.

Postoperative course

**Figure 4:**
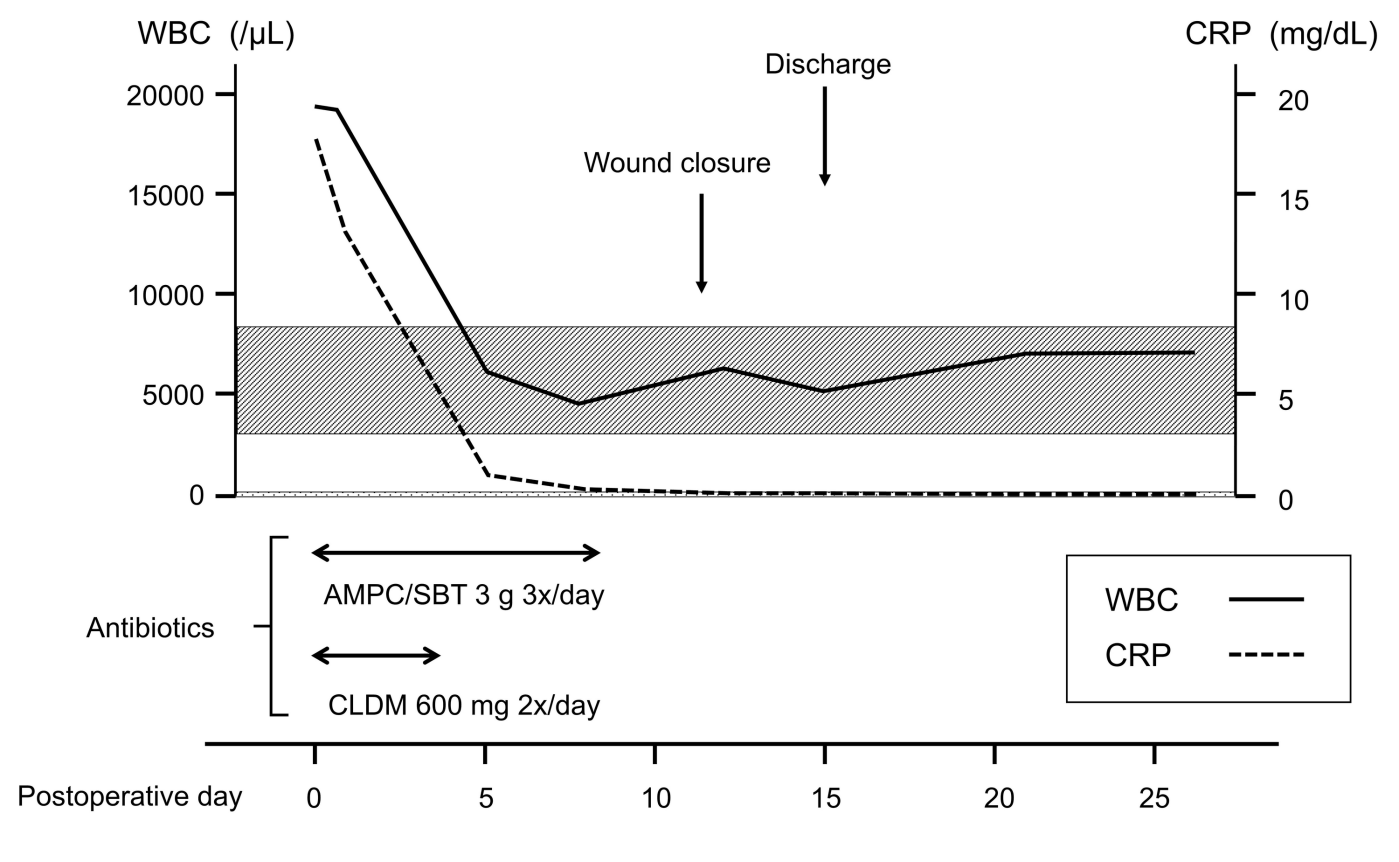
Postoperative course. The wound was managed with daily saline irrigation and intravenous antibiotics. By postoperative day 8, the inflammatory markers had normalized. The wound was closed on day 12, and the patient was discharged on day 15 with outpatient follow-up. The solid line in the graph represents the trend of the white blood cell count (WBC, ×10³/µL); the dotted line represents the trend of the serum C-reactive protein (CRP, mg/dL). The shaded area with diagonal lines indicates the normal range of WBC, and the shaded area with dotted lines indicates the normal range of CRP. The double-headed arrows indicate the duration of administration for each antibiotic. AMPC/SBT, ampicillin/sulbactam; CLDM, clindamycin

**Figure 5:**
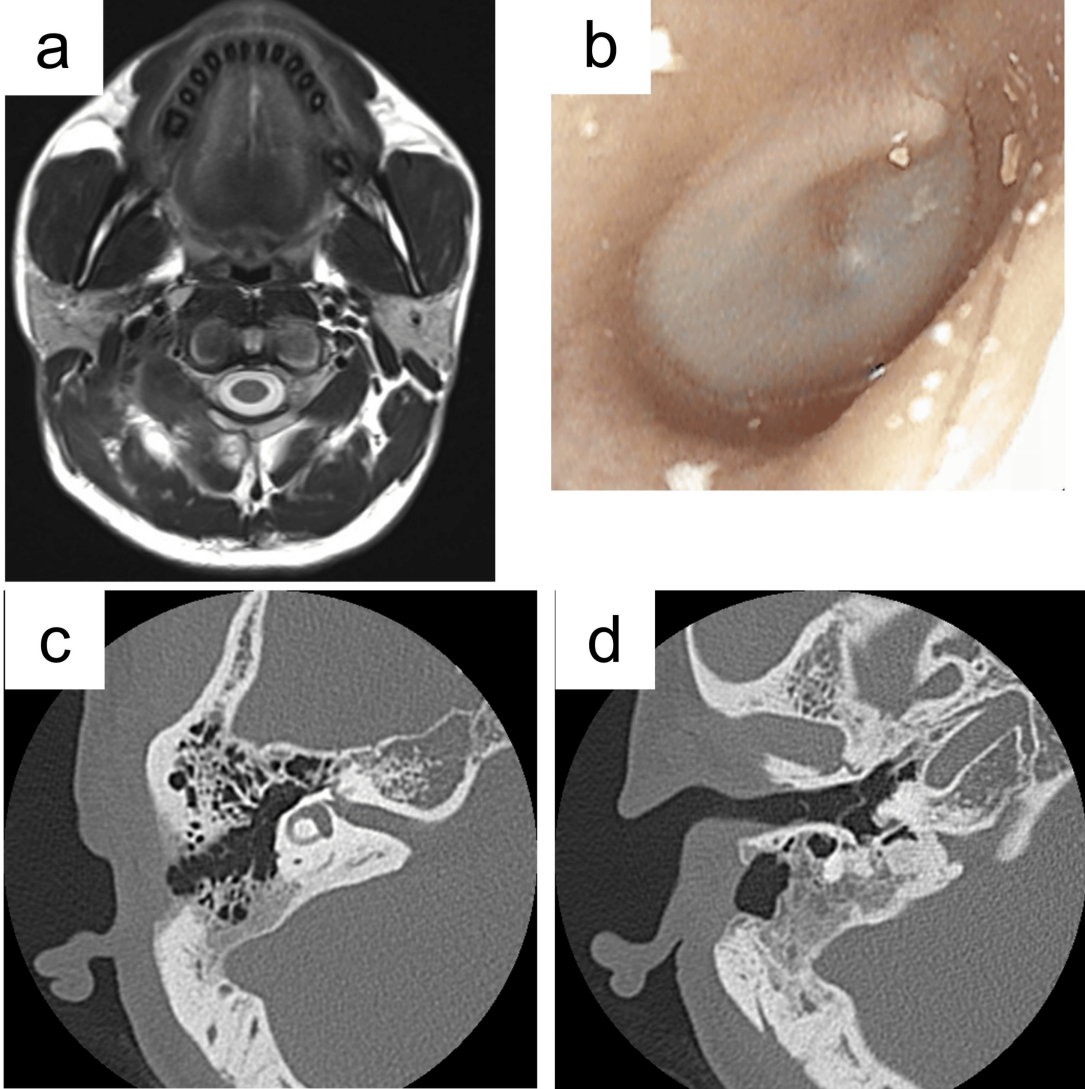
Follow-up at 1.5 and 6 months after surgery. (a) Axial T2-weighted MRI obtained 1.5 months postoperatively, demonstrating resolution of cervical muscle inflammation and collapse of the abscess cavity. (b) Otoscopic view of the right ear at 6 months postoperatively, showing resolution of external auditory canal swelling. The tympanic membrane remains slightly opaque but without erythema, bulging, or effusion. (c, d) Target CT of the temporal bone at 6 months postoperatively, demonstrating resolution of middle ear and mastoid opacification.

The patient’s postoperative recovery was uneventful (Figure 4, Table 1). Laboratory data on admission (operative day) demonstrated marked inflammation and hyperglycemia, both of which improved by postoperative day 15 (discharge). The wound was irrigated daily with saline through the drain. As no pathogens were detected on culture, antibiotic therapy with intravenous ampicillin/sulbactam (3 g three times daily for eight days) and clindamycin (600 mg twice daily for three days) was selected empirically for cervical abscess management. The inflammatory response (CRP) improved rapidly and normalized within one week. On postoperative day 12, the drain was removed and the wound was closed, and on day 15, the patient was discharged. During the hospitalization, routine blood testing revealed hyperglycemia, with a plasma glucose of 194 mg/dL, and an elevated HbA1c of 7.4%, confirming the diagnosis of diabetes mellitus, for which treatment was initiated by an endocrinologist. The patient was followed closely after discharge, and by postoperative day 20, the tympanic membrane had returned to normal (Figure 5a). Although the patient’s clinical symptoms had resolved, repeat CT and MRI were performed 1.5 months postoperatively to confirm complete resolution of the infection and exclude any residual inflammation. The imaging findings demonstrated full recovery of the middle ear and mastoid aeration and improvement of the surrounding soft tissue inflammation (Figures 5b-5c).

## Discussion

From masked mastoiditis to Bezold’s abscess

Bezold’s abscess is an extracranial complication of otitis media that results from the extension of infection through the mastoid tip into the sternocleidomastoid or digastric muscle sheath [6]. Although rare in the antibiotic era, the abscess still poses a diagnostic challenge, particularly when it complicates masked mastoiditis, a condition with minimal or absent otologic signs.

In our case, the patient had been treated repeatedly with short courses of antibiotics and exhibited only mild otologic symptoms, including intermittent otalgia, rather than the overt clinical presentation of acute otitis media or mastoiditis, before developing the cervical abscess. This clinical silence is consistent with masked mastoiditis, first described by Holt et al. [2] and subsequently by Marzal et al. [4], in which partial or inappropriate antibiotic therapy suppresses acute symptoms while allowing persistent, low-grade infection to progress within the mastoid cavity. Over time, this indolent infection may result in erosive bony destruction, enabling extratemporal spread [4]. Mastoiditis can be concealed by antibiotic administration and can be present without acute inflammatory signs, suggesting that inadequate or insufficient antibiotic use may contribute to its development [7]. Laboratory data on admission demonstrated marked neutrophil-predominant leukocytosis, elevated CRP, and hyperglycemia, indicating systemic inflammation and impaired glucose tolerance. These abnormalities improved postoperatively and after appropriate endocrinological management, consistent with the patient’s favorable clinical course.

Once the clinical silence of masked mastoiditis has been recognized, it is important to consider the anatomic conditions that facilitate its extension into the neck tissues.

Anatomical pathways and pneumatization as risk factors

The extension of infection into the cervical region depends on several anatomical factors. In well-pneumatized mastoids, pus may erode the medial aspect of the mastoid tip and escape into the digastric ridge and parapharyngeal space [8,9]. This pathway is facilitated by a large emissary foramen and low cortical resistance. In fact, pneumatization may paradoxically increase the vulnerability to extracranial spread, particularly in adults [10]. An additional report suggests that mastoid tip erosion is common and emphasizes the central role of CT in establishing the diagnosis [9].

In our case, CT imaging confirmed the presence of mastoid tip erosion and abscess formation beneath the sternocleidomastoid muscle, consistent with classic Bezold’s abscess. Importantly, the absence of middle ear perforation and the preserved tympanic membrane masked the underlying mastoiditis. Samuel et al. [3] reported intraoperative findings of granulation tissue filling the mastoid antrum and obstructing ventilation and drainage into the tympanum and mastoid air cells. This mechanism may have contributed to our patient’s disease progression as well, given that our operative findings similarly demonstrated granulation tissue occupying the mastoid cavity and impeding adequate drainage.

Diagnostic challenges and imaging

Masked mastoiditis complicates the timely diagnosis of Bezold’s abscess. Symptoms such as otalgia, otorrhea, and hearing loss may be absent, leading to a false sense of resolution after incomplete antibiotic treatment. Imaging thus becomes paramount.

CT scanning is the gold standard for detecting cortical bone erosion and opacification of the mastoid air cells. In our case, axial and coronal views revealed characteristic tip erosion and adjacent fluid collection. MRI may be useful to evaluate soft tissue extension and exclude intracranial involvement [11].

Following these diagnostic considerations, it is essential to examine the microbiological background. 

Microbiology and antibiotic history

No pathogens were isolated from either the mastoid cavity specimen or the cervical abscess cultures; this is not uncommon in patients who have received antimicrobial therapy. Previous reports suggest that streptococci, including *S. pneumoniae* and *viridans* group species, remain among the most frequently implicated organisms [12], whereas anaerobes and polymicrobial flora are more commonly reported in chronic otitis media with cholesteatoma [13]. However, repeated short courses of antibiotics, as seen in our patient, can suppress bacterial growth and contribute to negative culture results. This underscores the challenge of correlating microbiological data with clinical progression in masked mastoiditis, because prior antibiotic use may also reduce otologic symptoms without eradicating infection.

Beyond microbiology, a comparison with previously reported cases provides clinical context.

Comparative case literature

Several case reports and reviews corroborate our findings and further illustrate the spectrum of Bezold’s abscess presentations. These reports not only emphasize the diagnostic challenges but also highlight the diversity of clinical contexts in which this complication may arise. A structured overview is presented in Table 2.

**Table 2:** Comparative case reports and literature review on Bezold’s abscess. Summary of previously reported cases and reviews relevant to Bezold’s abscess. Each entry highlights the patient context, key findings, and clinical relevance. This table was independently created by the authors by compiling data from the cited sources.

Author (Year)	Patient/Context	Key findings	Clinical relevance
Uchida et al. (2002) [14]	Review of 18 Japanese cases, including one recurrent cholesteatoma 20 years after surgery	Demonstrated variability of clinical contexts and long-term risk	Highlights the importance of vigilance even decades after the initial disease
Lin et al. (2015) [6]	Patient with Bezold’s abscess without overt otitis	Highlighted the role of imaging in diagnosis	Imaging is essential even when ear symptoms are absent
Correia-Rodrigues et al. (2021) [8]	Case of deep neck abscess complicating otomastoiditis	Emphasized early suspicion in unclear deep neck abscesses	Early otologic evaluation is warranted
Young et al. (2022) [15]	Review of the aging immune system	Pointed out missed mastoid pathology in URI patients	Immunosenescence may predispose to atypical progression
Alkhaldi et al. (2022) [11]	Case mimicking a parapharyngeal space tumor	Demonstrated diagnostic ambiguity	Deep neck masses may conceal an otogenic origin
Ansari et al. (2024) [1]	Recent case report	Showed the ongoing relevance of Bezold’s abscess despite its rarity	Confirms contemporary clinical occurrence

Collectively, these studies, including those by Uchida et al. [14], Lin et al. [6], Alkhaldi et al. [11], Correia-Rodrigues et al. [8], Young et al. [15], and Ansari et al. [1], reinforce that Bezold’s abscess, though rare, continues to be encountered across diverse clinical settings. These cases, like ours, emphasize that a deep neck abscess of unknown origin warrants prompt imaging of the temporal bone, especially in elderly or immunocompromised patients.

Consideration of management strategies follows logically from clinical parallels.

Therapeutic considerations

Laboratory data on admission (operative day) demonstrated marked neutrophil-predominant leukocytosis, elevated CRP, and hyperglycemia, indicating systemic inflammation and impaired glucose tolerance. These abnormalities improved postoperatively and after appropriate endocrinological management (postoperative day 15, discharge), consistent with the patient’s favorable clinical course.

The management of Bezold’s abscess requires a two-pronged approach comprising: (1) surgical drainage of the cervical abscess, typically via a transcervical approach, and (2) mastoidectomy to address the infectious nidus. In our case, a canal wall-up mastoidectomy was sufficient, given the absence of cholesteatoma or extensive necrosis, with empirical broad-spectrum antibiotics to target both aerobic and anaerobic organisms. Upon identification of specific pathogens, therapy should be tailored. In addition, surgical management must include both drainage of the cervical abscess and careful removal of obstructing granulation tissue within the mastoid cavity to restore adequate ventilation and drainage and to achieve durable resolution.

Immunologic and host factors

Bezold’s abscess is often associated with immunocompromised states, and our patient was no exception. Indeed, his previously undiagnosed diabetes mellitus became evident during the current illness. Hyperglycemia impairs neutrophil chemotaxis, phagocytosis, and microbial killing, predisposing individuals to deep-seated infections [14]. The silent progression of masked mastoiditis and the delayed immune response in this case may be attributable in part to impaired host defenses from undetected metabolic dysfunction.

Diabetes has been identified in multiple reports as a risk factor for both the development and the progression of deep neck infections and otogenic complications. Lin et al. and Rodriguez et al. also identified host metabolic status as a contributing factor in the evolution of atypical presentations [6,10]. Therefore, early evaluation for metabolic and immunological disorders should be considered in adult patients presenting with deep neck infections of unclear etiology. Recognizing diabetes when patients present with such infections may influence both immediate management and long-term health outcomes.

## Conclusions

This case reaffirms the fact that masked mastoiditis remains an underrecognized but clinically important precursor to Bezold’s abscess. Clinicians should maintain a high index of suspicion for otogenic sources in patients with deep neck infections, particularly in those who are elderly or those with recent upper respiratory infections and prior antibiotic use. The identification of previously undiagnosed diabetes mellitus in our patient further highlights the role of host immune status. Prompt imaging, early surgical intervention, and mastoid exploration remain critical to favorable outcomes.
